# Emerging integrated nanoclay-facilitated drug delivery system for papillary thyroid cancer therapy

**DOI:** 10.1038/srep33335

**Published:** 2016-09-12

**Authors:** Yi Zhang, Mei Long, Peng Huang, Huaming Yang, Shi Chang, Yuehua Hu, Aidong Tang, Linfeng Mao

**Affiliations:** 1 Centre for Mineral Materials, School of Minerals Processing and Bioengineering, Central South University, Changsha 410083, China; 2 Xiangya Hospital, Central South University, Changsha 410078, China; 3 Hunan Key Lab of Mineral Materials and Application, Central South University, Changsha 410083, China; 4 State Key Lab of Powder Metallurgy, Central South University, Changsha 410083, China; 5 School of Chemistry and Chemical Engineering, Central South University, Changsha 410083, China

## Abstract

Nanoclay can be incorporated into emerging dual functional drug delivery systems (DDSs) to promote efficiency in drug delivery and reduce the toxicity of doxorubicin (DOX) used for thyroid cancer treatment. This paper reports the expansion of the basal spacing of kaolinite nanoclay was expanded from 0.72 nm to 0.85 nm, which could provide sufficiently spacious site for hosting doxorubicin molecules and controlling the diffusion rate. A targeted design for papillary thyroid cancer cells was achieved by introducing KI, which is consumed by the sodium-iodide symporter (NIS). As indicated by MTT assays, confocal laser scanning microscopy and bio-TEM observations, methoxy-intercalated kaolinite (Kaolin_MeOH_) exhibited negligible cytotoxicity against papillary thyroid cancer cells. By contrast, DOX-Kaolin_MeOH_ showed dose-dependent therapeutic effects *in vitro*, and KI@DOX-Kaolin_MeOH_ was found to act as a powerful targeted therapeutic drug. Furthermore, active and passive targeting strategies played a role in the accumulation of the drug molecules, as verified by an *in vivo* bio-distribution analysis.

The anticancer drug doxorubicin (DOX), an anthracycline antibiotic with the trade name Adriamyacin, is a well-established drug that is effective against various types of cancers. The drug’s effective anti-tumour activity involves DNA intercalation, topoisomerase II inhibition, and free radical formation. However, DOX causes various negative effects, such as alopecia, myelosuppression, nausea, vomiting, and oral mucositis. To increase the efficiency of drug delivery and reduce DOX toxicity, drug delivery systems (DDSs) with targeted designs have been introduced. Advanced DDSs should be synthetically straightforward and biocompatible, and exhibit biodegradability and targeting behavior^1^,^2^,^3^. The complex manufacturing procedures for such systems often involve large amounts of toxic chemicals (particularly organic reagents and strong acids/alkalis) and are time-consuming and costly^4^,^5^,^6^,^7^,^8^. Biocompatible composites are typically designed to be non-biodegradable, durable and mechanically stable. The optimal particle size for these composites is between 10 and 200 nm, and their zeta potential should be negative^9^,^10^. Targeting strategies should enable the accurate delivery of drugs to the desired sites and ensure the complete excretion of the carrier after treatment^5^,^11^. Thyroid cancer is one of the most common malignant neoplasms; it exhibits complicated processes^12^,^13^,^14^, and significant negative effects can result from most methods of thyroid cancer treatment, including surgical removal, radioactive iodine therapy, external beam radiotherapy, chemotherapy, and gene therapy^15^,^16^,^17^,^18^. Thyroid follicular epithelial cells exhibit a strong iodine uptake capacity, and the iodine compounds they absorb are the primary material used by the thyroid to synthesize thyroid hormones after oxidization^19^,^20^,^21^. A number of targeted DDSs based on carrier modification, drug molecule loading and the assembly of specific targeting assembly have been used in thyroid cancer treatment^22^,^23^,^24^,^25^,^26^.

As efficient and low-toxicity drug carriers, nanoclay minerals can induce stronger therapeutic effects by providing higher and longer lasting drug concentrations by virtue of their adsorption capacity, ion exchange property, biostability, degradability and excellent biocompatibility^5^,^27^,^28^,^29^,^30^,^31^,^32^,^33^. As one of the three traditional Chinese medicinal materials, nanoclay has been widely used in medical practices since prehistory. During the Ming Dynasty (1368–1644 AD), the great medical scientist Shizhen Li wrote the Compendium of Materia Medica, in which he recorded a detailed description of each clay mineral’s name, source, shape, color and method of collection. With the recent development of nano-biotechnology, clay minerals, such as halloysite, montmorillonite and kaolinite, have attracted increasing interest for use in biomedical related applications^34^,^35^,^36^,^37^,^38^,^39^.

In this study, we used methoxy-intercalated kaolinite (Kaolin_MeOH_) as a typical nanoclay container for the targeted delivery of DOX to cancer tissues with correspondingly diminished its side effects. Kaolinite (Kaolin, Al_2_Si_2_O_5_(OH)_4_) is a 1:1 two-dimensional aluminosilicate consisting of octahedral gibbsite Al(OH)_3_ and tetrahedral SiO_4_ sheets^40^,^41^. Most active agents (e.g., salicylic acid and ibuprofen) can be entrapped within its inter-particle spaces and only adsorbed on the external surfaces instead of retained in the inter-layer space^42^. Methoxy intercalation is a practical method for increasing the interlayer distance of this material from its original value of 0.72 nm to 0.85 nm, thus making the inter-layer space sufficiently large to accommodate molecules of drugs^43^,^44^,^45^,^46^ such as DOX (0.28~0.84 nm). Thus, the excellent loading capacity and controlled-release properties of Kaolin_MeOH_ can be successfully exploited for DOX delivery. To specifically target thyroid cancer cells, in this study, nanocomposites of this kind were coated with KI. In addition to the procedures described above, PEGylation was also applied to improve the permeability, stability and anti-corrosion of these nanocomposites *in vivo* and to minimize macrophage phagocytosis. We believe that this combination of techniques is highly meaningful because it can give rise to an effective targeted DDS for thyroid cancer treatment (Fig. 1).

## Results

**Figure 2:**
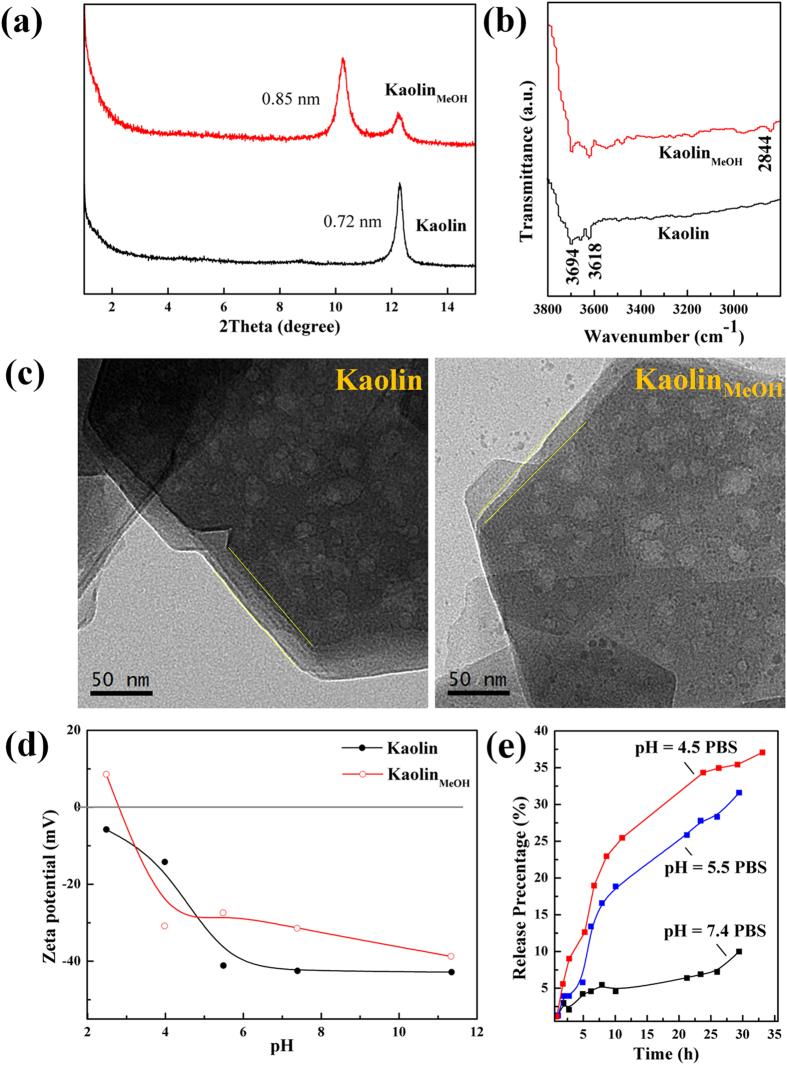
Material characterization. (**a**) XRD patterns, (**b**) DRIFT spectra, (**c**) TEM images, and (**d**) zeta curves of the Kaolin samples. (**e**) DOX release profiles for KI@DOX-Kaolin_MeOH_ at various pH values (pH = 7.4, 5.5 and 4.5).

Tests of the design described above revealed that this targeted KI@DOX-Kaolin_MeOH_ DDS is highly effective for papillary thyroid cancer treatment. Thus, the features introduced by the simple manufacturing procedure should be discussed. The peaks observed for original kaolinite were similar to the characteristic data for kaolinite (JCPDS card No. 14-0164, Figure S1a), with a characteristic d_001_ value of 0.72 nm (Fig. 2a), whereas the Kaolin_MeOH_ exhibited two characteristic d_001_ values of 0.85 and 0.72 nm, corresponding to the kaolinite with intercalated methoxy groups and the residual un-intercalated kaolinite, respectively. The methoxy-modified kaolinite was prepared by first intercalating dimethyl sulfoxide (DMSO) into the interlayer space of the kaolinite. The DRIFT spectra of Kaolin and Kaolin_MeOH_ are presented in Fig. 2b and Figure S1. For Kaolin, the peak at 3694 cm^−1^ was assigned to the O-H stretching vibrations of the inner-surface Al-OH groups, whereas the peak at 3618 cm^−1^ was ascribed to the O-H stretching vibrations of the inner Al-OH groups (at the interface of the Si-O tetrahedron and the Al-O octahedron). For Kaolin_MeOH_, the peak at 2848 cm^−1^ was attributed to the C-H stretching vibrations of the methoxy groups. The particle size and zeta potential of the carriers were also characterized, as the size and charge of carriers can affect their uptake by cells and their distribution in organs (Fig. 2c,d). The original kaolinite exhibited a typical pseudo-hexagonal morphology with a diameter of 150 to 200 nm. The distance between adjacent layers was loose after methoxy modification, whereas the platy morphology did not change. The zeta potential of the kaolinite was negative over the entire pH range region, ranging from −5.5 mV (pH 2.5) to −43 mV (pH 11). For an acidic pH of approximately 2.5, the zeta potential of Kaolin was approximately −5.5 mV. As the pH was increased to pH 5.5, this value rapid decreased. Finally, the zeta potential slowly declined from −41 to −43 mV as the pH was increased to 11.5. For Kaolin_MeOH_, the zeta potential ranged from 8.5 mV (pH 2.5) to −38 mV (pH 11). The isoelectric point was at approximately 2.8, and the zeta potential was slightly more positive than that of Kaolin, with a value of −30 mV in the neutral solution. Based on the basal spacing calculations, the surface morphology analysis and the zeta potential results, it can be deduced that Kaolin_MeOH_ exhibits a negative surface charge and unsatisfied valences resulting from the broken bonds at the crystal edges (positive charges at low pH and negative charges at high pH), which could promote more interactions between organic molecules and particle-particle spaces or inter-layer space involving various mechanisms, including hydrogen bonding, ion exchange and hydrophilic/hydrophobic interactions. The inter-layer space was expanded by the intercalated methoxy molecules, allowing the loading capacity for positively charged DOX to reach 54.52 wt % (Table S1). The differences in the *in vitro* drug release patterns observed at pH values of 7.4, 5.5 and 4.5 primarily arose from differences in the electrostatic interactions between the positively charged DOX molecules and the oppositely charged Kaolin surface^22^. The controlled release behaviours of DOX-Kaolin_MeOH_ in PBS solutions with different pH values are presented in Fig. 2e and Table S2. Under simulated normal physiological conditions (pH 7.4), DOX-Kaolin_MeOH_ exhibited a very low DOX release rate, with a cumulative release of 9.5% over 30 h, which could be attributed to the strong electrostatic interactions between the positively charged DOX molecules and the oppositely charged Kaolin surfaces at neutral pH. Under simulated tumour intracellular conditions (pH 5.5), DOX-Kaolin_MeOH_ showed a faster DOX release rate, with a cumulative release of 32.5% over 30 h, which could be related to the reduced electrostatic interaction resulting from the increased charge of the Kaolin_MeOH_ with the decrease in pH. This pH-responsive controlled release behaviour could be useful for promoting targeted drug delivery to cancer tissues while diminishing the drug’s side effects, considering that the blood is neutral (pH 7.4), tumour extracellular conditions are acidic (pH 6.5) and the endosome-lysosomes conditions are more strongly acidic (pH 5.0~5.5)^47^.

**Figure 3:**
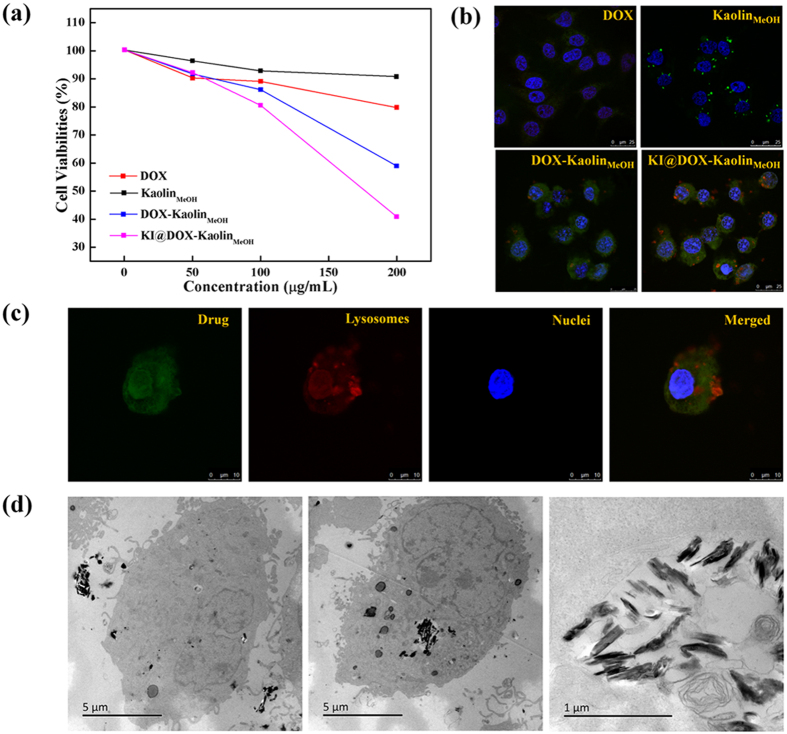
Cytotoxicity evaluation. (**a**) Cytotoxicity after incubation with free DOX, Kaolin_MeOH_, DOX-Kaolin_MeOH_ and KI@DOX-Kaolin_MeOH_ at different concentrations as assessed via the MTT protocol. (**b**) CLSM images of papillary thyroid cancer cells after the uptake of free DOX, Kaolin_MeOH_, DOX-Kaolin_MeOH_, or KI@DOX-Kaolin_MeOH_. (**c**) CLSM images of papillary thyroid cancer cells incubated with KI@DOX-Kaolin_MeOH_ at an equivalent DOX concentration of 10 μg/mL for 4 h. (**d**) Bio-TEM images of papillary thyroid cancer cells after incubation with KI@DOX-Kaolin_MeOH_.

Cell proliferation experiments were performed to demonstrate the therapeutic efficacy of DOX-Kaolin_MeOH_ for *in vitro* papillary thyroid cancer treatment, despite the vast literature describing the excellent biostability, biocompatibility and degradability of nanoclay minerals against cancer cells *in vitro* and *in vivo*. Regarding the cytotoxicity against papillary thyroid cancer cells *in vitro* (Fig. 3a and Figure S2), the observed effects of cell death and growth inhibition could be predominantly attributed to DOX, whereas Kaolin_MeOH_ showed a very low cytotoxic effect. Free DOX (>100 μg/mL) exhibited little cytotoxicity effects against papillary thyroid cancer cells, but no obviously increase, which could result from the poor water solubility (50 mg/mL, water). DOX-Kaolin_MeOH_ exhibited obvious inhibiting effects on cellular growth as the nanocomposite concentration increased. Actually, Kaolin_MeOH_, as an efficient and low-toxicity drug delivery carrier, could increase the water solubility of DOX in tissues, facilitate intimate contact with cancer cell membrane and controlled release of DOX in cancer tissue, and decrease the distribution of DOX in normal tissue due to its adsorption capacity, ion exchange property, biostability, degradability and excellent biocompatibility. However, KI@DOX-Kaolin_MeOH_ induced even the highest levels of cell death compared with the equivalent doses of free DOX at high concentrations in a dose-dependent manner, which could be attributed to the targeting function of KI. The internalization of KI@DOX-Kaolin_MeOH_ into cancer cells and the subsequent intracellular drug release behaviour was investigated via confocal laser scanning microscopy (CLSM) (Fig. 3b and Figure S3). In these figures, lysosomes, nanocomposites and nuclei were labelled in red, green and blue, respectively. The nanocomposites could not pass through the nuclear membranes, as indicated by yellow or yellow-green signals in the images (a yellow signal arises from a mixture of red and green). In the confocal microscopy image of thyroid cancer cells incubated with KI@DOX-Kaolin_MeOH_, yellow and yellow-green signals are evident within the intracellular regions and around the blue nucleus (Fig. 3c), suggesting that some nanocomposites entered the lysosomes, while the remainder were located in the cytoplasm. Further information about the cellular uptake mechanism and the intracellular location of the KI@DOX-Kaolin_MeOH_ in the papillary thyroid cancer cells was revealed through a bio-TEM analysis (Fig. 3d). KI@DOX-Kaolin_MeOH_ was observed in the cytoplasm and within the vesicles near the cell membrane, indicating that KI@DOX-Kaolin_MeOH_ was internalized through endocytosis, i.e., through the formation of vesicles that then passed through the cell membrane.

**Figure 4:**
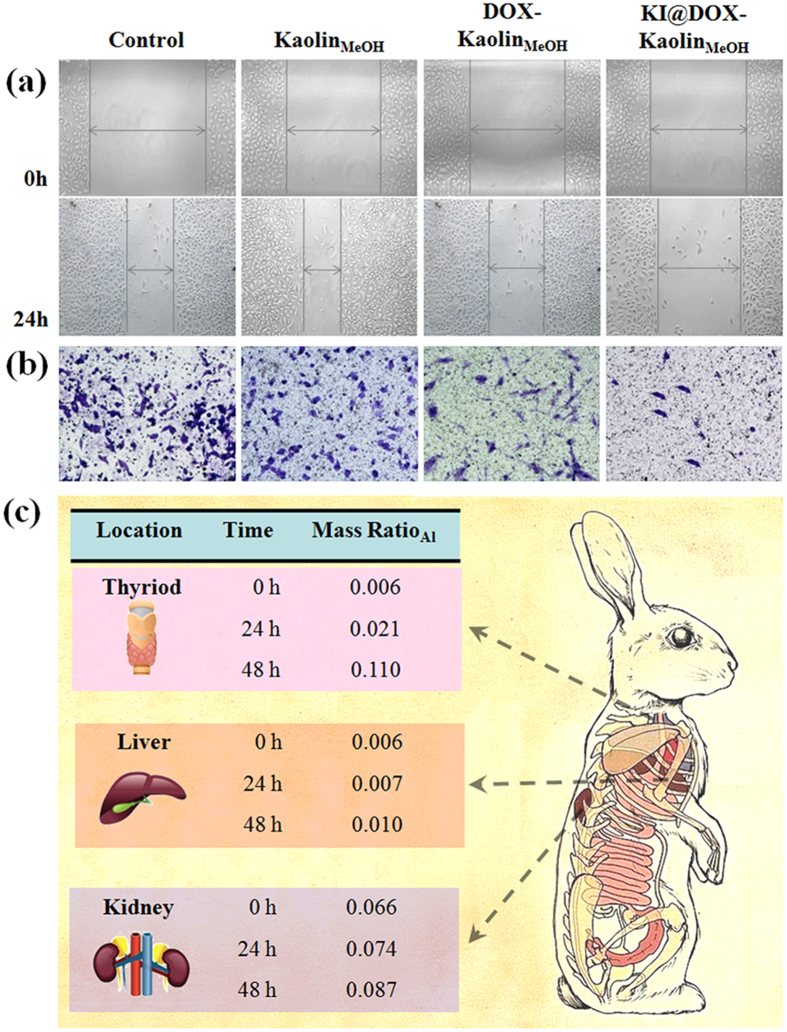
Anti-metastasis assays. (**a**) Inhibition of the migratory potential of papillary thyroid cancer cells as indicated by wound healing assays before (above) and after (below) the different treatments (control, Kaolin_MeOH_, DOX-Kaolin_MeOH_ and KI@DOX-Kaolin_MeOH_). (**b**) Transwell migration assay of papillary thyroid cancer cells after treatment with different therapeutic agents (control, Kaolin_MeOH_, DOX-Kaolin_MeOH_ and KI@DOX-Kaolin_MeOH_): representative microscopic images of the migrated papillary thyroid cancer cells. (**c**) Bio-distribution of KI@DOX-Kaolin_MeOH_ in various organs (thyroid gland, liver, kidney) after 24 h and 48 h (right).

The cell proliferation experiments described above demonstrated that DOX-Kaolin_MeOH_ exhibited dose-dependent therapeutic effects *in vitro*, and that its performance was further optimized by the KI coating by virtue of the resultant targeting capability. Anti-metastasis was evaluated through migration and wound healing assays. In the wound healing assays, microscopic images (Fig. 4a) showed that the anti-wound healing activities of DOX and Kaolin_MeOH_ resulted in behaviour similar to that of untreated cells, whereas KI@DOX-Kaolin_MeOH_ almost completely inhibited repair of the wounded area. In the migration assay (Fig. 4b and Figure S4), KI@DOX-Kaolin_MeOH_ exhibited a excellent inhibition compared with papillary thyroid cancer cells incubated with DOX, Kaolin_MeOH_ or DOX-Kaolin_MeOH_. The invasiveness of the papillary thyroid cancer cells after incubation with DOX-Kaolin_MeOH_ and KI@DOX-Kaolin_MeOH_ was decreased by approximately 88% and 72% (Figure S4), respectively. These excellent *in vitro* results suggested the feasibility of obtaining the same results *in vivo*; however, more restrictive requirements apply in the latter case provided, such as biostability, degradability and excellent biocompatibility (Figure S5). The bio-distribution of KI@DOX-Kaolin_MeOH_ in various organs (thyroid gland, liver, kidney) was investigated by using inductively coupled plasma atomic emission spectroscopy (ICP-AES) to determine the amount of Al present due to the intrinsic *in vivo* Al background (Fig. 4c). After 24 and 48 h of administration, the treated animals were euthanized, and the amount of Al in the selected organs were detected. As expected, KI@DOX-Kaolin_MeOH_ accumulation was evident in the thyroid gland, and the level of accumulation had increased 3-fold and 4-fold after 24 and 48 h, respectively. By contrast, the accumulation in the liver and kidneys was time-independent, exhibiting no obvious growth, thus implying that KI@DOX-Kaolin_MeOH_ can be used for efficient, targeted, and safe cancer therapy.

## Discussion

**Figure 5:**
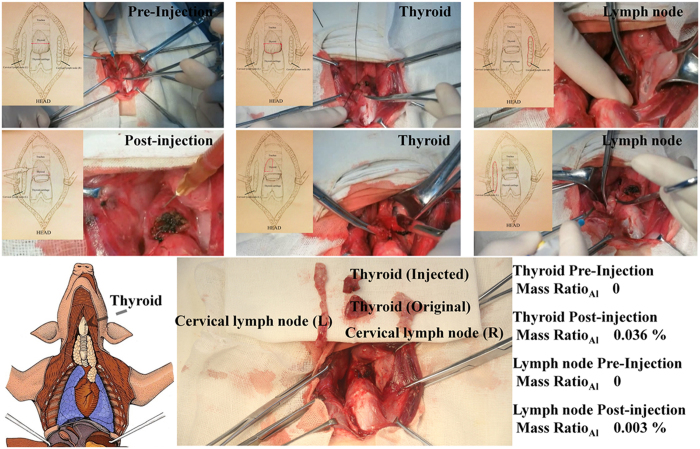
Orthotopic injection. Mass ratio of Al in thyroid and lymphnodesbefore and after KI@DOX-Kaolin_MeOH_ injection.

Passive targeting was verified by the direct injection of drugs into the thyroid glands of pigs. The accumulation of KI@DOX-Kaolin_MeOH_ in the thyroid and lymph nodes was detected based on the ICP-AES result for Al. The mass ratio of Al in the thyroid was 0.036% after an orthotopic injection of 100 μL of 10 mg/mL KI@DOX-Kaolin_MeOH_ (Fig. 5). A small amount of KI@DOX-Kaolin_MeOH_ infiltrated the lymph nodes because of the passive targeting, and the resulting mass ratio of Al was 0.003%.

**Figure 6:**
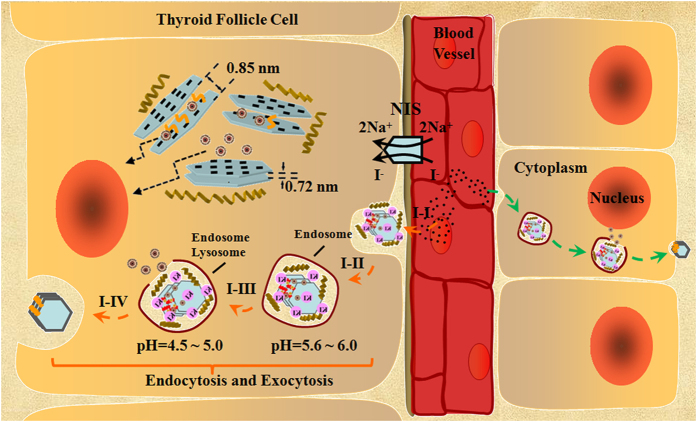
Delivery mechanism. Schematics illustration of the mechanismof the intracellular delivery of DOX by KI@DOX-Kaolin_MeOH_.

A mechanism was proposed to describe the action of the KI@DOX-Kaolin_MeOH_ DDSs against papillary thyroid cancer cells; this mechanism is schematically illustrated in Fig. 6. At the cellular level, these targeted DDS agents are intravenously implanted into the blood vessel and immediately internalized through active targeting and endocytosis. The endocytosis process included the internalization of the targeted DDS agents in the endosomes, localization in the endo-lysosomes, and the release and accumulation of DOX in the cell nucleus. The active targeting ensured that more drug molecules reached the desired site following an iodide uptake process by the sodium-iodide symporter (NIS), which mediates the efficient iodide uptake from the bloodstream into the thyroid. Furthermore, more DOX is released from the targeted DDS agents in the acidic microenvironment of cancer cells than in macrophages because of the larger size and increased amount of acidic metabolic products in cancer cells compared with normal cells.

In summary, agents of an emerging Kaolin_MeOH_-based targeted DDSs were prepared using a DMSO-treated kaolinite intercalation complex as an intermediate by combining the advantages of the targeting behaviour of KI with DOX chemotherapy for papillary thyroid cancer treatment. Drug release from the inter-layer space of methoxy-intercalated kaolinite exhibited different controlled release behaviours at different pH values. The KI targeting capability enhanced the therapeutic effect by virtue of the iodide uptake process of the NIS, which is observed in papillary thyroid cancer cells but not in macrophages. CLSM and bio-TEM observations revealed that DDS agents were internalized through endocytosis and exhibited dose-dependent therapeutic effects *in vitro*. Importantly, DOX-Kaolin_MeOH_ exhibited obvious inhibition *in vitro*, and its performance was further optimized by the KI coating. The outstanding results provide new insight at the intersection of biomaterials science and biomedical fields and support the expectation that nanoclay will to be a promising platform for emerging methods of tumour-targeting drug delivery.

## Methods

### Synthesis of KI@DOX- Kaolin_MeOH_ and its release behaviour *in vitro*

#### Methoxy intercalated kaolinite

First, dimethyl sulfoxide (DMSO) intercalated kaolinite was synthesize by dispersing kaolinite into neat liquid DMSO at 60 °C for 12 h. Second, 5.0 g of the aforementioned nanocomposites was dispersed in 100 mL methanol solution at room temperature for 7 days. Then, the mixture was separated, stored and labelled as Kaolin_MeOH_.

#### KI assembly

50 mg DOX-Kaolin_MeOH_ was dispersed in 30 mL of 8 mg/mL KI solution under dark conditions for 3 h. Then, the mixture was separated, stored and labelled as KI@DOX-Kaolin_MeOH_.

#### PEGylation

KI@DOX-Kaolin_MeOH_ was dispersed in100 mL of an ethanol solution containing 1 mL methoxy PEG silane (M-PEG-SLN MW 5000) and refluxed at 80 °C for 24 h. Then, the mixture was separated, washed, stored and labelled as PEGylated KI@DOX- Kaolin_MeOH_.

#### DOX loading and release in vitro from DOX-Kaolin_MeOH_ at various pH values (pH = 7.4, 5.5 and 4.5)

5 mg Kaolin_MeOH_ was dispersed in 6 mL of 0.5 mg/mL DOX PBS solution under dark conditions for 24 h. Then, the mixture was separated, stored and labelled as DOX-Kaolin_MeOH_. The loading rate was evaluated by the measurement of the residual DOX solution based on the UV-vis spectra at a wave length of 480 nm. For assessment of *in vitro* DOX release, 2 mg of DOX-Kaolin_MeOH_ was first packaged in a dialysis bag and then placed into 15 mL volumes of various PBS solutions with different pH values (pH = 7.4, 5.5 and 4.5). The release rates were monitored based on the UV-vis spectra over time.

### *In vitro* cytotoxicity

#### MTS assay

*In vitro* cytotoxicity and cell proliferation were evaluated via MTS reduction assays. The papillary thyroid cancer cell line TPC1, which is able to actively uptake iodide, was used in this study. TPC-1 cells were firstly incubated with RPMI-1640 at 37 °C in 5% CO_2_ for 24 h, and a fresh medium containing the nanocomposites (0, 50, 100, 200 μg/mL) was then used for further incubated for 24 h. Then, 20 μL of the MTS working solution was added per well and incubated for 3 h before a colorimetric readout was performed at 490 nm. The cytotoxicity was assessed based on the percentage cell viability compared with that of untreated control cells. Finally, the nuclei were stained with DAPI and observed using a fluorescence microscope (Leica,Germany).

#### Bio-TEM observations

TPC-1 cells were firstly incubated with RPMI-1640 at 37 °C in 5% CO_2_ for 24 h, and a fresh medium containing the nanocomposites (0, 50, 100, 200 μg/mL) was then used for further incubation for 24 h. Subsequently, the cells were washed, detached and embedded for viewing via JEM-1230 electron microscopy.

#### Confocal laser scanning microscopy (CLSM) observations

TPC-1 cells were first incubated in a CLSM-special culture dish at 37 °C in 5% CO_2_ unitl a cell density of 60~70% was achieved, and fresh medium containing the nanocomposites (2 mL, 50 μg/mL) was then used for further incubation for 4 h. Then, the cells were washed, detached and nuclear staining with DAPI was performed for viewing with CLSM (SP8, Leica, Germany)

#### Wound healing, cell migration and invasion assays

For the wound healing assay, TPC-1 cells were first incubated with RPMI-1640 at 37 °C in 5% CO_2_ for 24 h and then washed and detached. The confluent cell monolayers were wounded using a uniform scrape and fresh medium containing the nanocomposites was used for further incubation for 24 h. The wound closure was monitored via optical microscopy (Olympus, Jan), and images of the wound were captured immediately and at 24 h post-scratching. For the cell migration assay, TPC-1 cells were first incubated with RPMI-1640 at 37 °C in 5% CO_2_ for 24 h, and fresh medium containing the nanocomposites was then used for further incubation for 24 h. Then, the cells were harvested, fixed and stained with 0.1% crystal violet. The cells were added to the upper compartments of a polycarbonate filter (pore size, 8 μm) and counted on the lower compartments using microscope. The relative rate of migration was calculated as follows: Migration%=(migrating cells with the treatment/migrating cells without treatment)×100%.

#### High-content screening (HCS)

Human papillary thyroid cancer (TPC-1), human anaplastic thyroid cancer (FRO), human hepatocellular cancer (HepG2), human lung cancer (A549), human pancreatic cancer (BXPC-3), human gastric cancer (MGC-803), human prostate cancer (T24), human breast cancer (MCF-7), human cervical cancer (HeLa), human colon cancer (LOVO), human esophageal cancer (EC9706), and mouse macrophages cells (RAW264.7) were firstly incubated with RPMI-1640 at 37 °C in 5% CO_2_ for 24 h, and fresh medium containing FITC-grafted nanocomposites (FITC-Kaolin_MeOH_ and FITC-DOX-Kaolin_MeOH_, 2 mL, 50 μg /mL) was then used for further incubation for 24 h. Then, the cells were washed and detached, and nuclear staining with DAPI was performed for viewing via high-content screening (Operetta, PerkinElmer, America). The intensity of green fluorescent protein (EGFP) was detected as indicative of the amount of drug intake.

### *In vivo* experiments

All experiments were performed in accordance with relevant guidelines and regulations of Central South University, and all experimental protocols were approved by the Hunan Provincial Department of Science and Technology, China. The animals used in this study were obtained from the animal laboratory of Central South University (Hunan, China). The animals were maintained in individual clean cages, and the research procedures were approved by the Institutional Animal Care and Use Committee.

#### Passive targeting research in vivo (mini-pig)

Female mini-pigs (weight range: 18~20 kg) were anesthetized with 3% sodium pentobarbital by means of a 1 mL/kg dose via an ear vein injection, at a rate of 2 mL/min. The surgical procedure used was modified radical neck dissection for thyroid cancer. An anterior median longitudinal incision was made with a length of approximately 8 cm. Skin and subcutaneous tissues were opened using a sharply instrument, and bleeding vessels were cauterized. The neck muscles layers were successively cut to free the thyroid and bilateral cervical lymph nodes. After the thyroid gland was divided into left and right lobes, the right lobe and the right cervical lymph node were resected as the control group. Then, 200 μL Kaolin_MeOH_ (10 mg/mL) was injected into the residual thyroid gland. 10 minutes after injection, part of left lobe and the left cervical lymph node were resected as the test group. Finally, the muscles and skin were layered and sutured with 3–0 nylon. The details are presented in a video provided in the Supporting Materials. The mini-pigs tolerated the procedure very well and were sent to recovery in stable condition. Surgical specimens were subjected to an aluminium (Al) element detection via inductively coupled plasma atomic emission spectroscopy (ICP-AES), and the Al element content was treated as representative of the amount of Kaolin_MeOH_ spread to the tissues.

#### Active targeting research in vivo (rabbit)

Bio-distribution experiments were performed on female Japanese white rabbits, which were fed a no-iodine diet for 15 days. Samples were implanted into the blood vessel through ear intravenous administration for 24 h and 48 h. The animals were euthanized, and the organs were used for further analysis. ICP-AES was used to measure the Al ion concentration used to indirectly verify the bio-distribution data of KI@DOX-Kaolin_MeOH_.

## Additional Information

**How to cite this article**: Zhang, Y. *et al*. Emerging integrated nanoclay-facilitated drug delivery system for papillary thyroid cancer therapy. *Sci. Rep*. **6**, 33335; doi: 10.1038/srep33335 (2016).

## Supplementary Material

Supplementary Information

Supplementary Vidoe S1

## Figures and Tables

**Figure 1:**
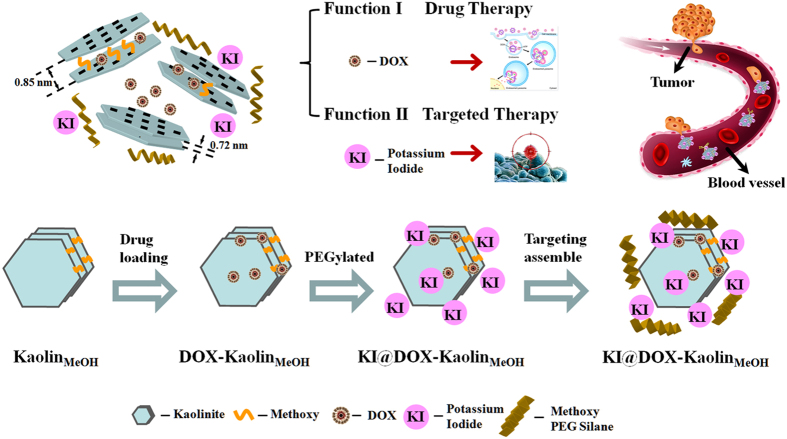
Material synthesis procedure. Schematic diagram of the procedure for synthesizing KI@DOX-Kaolin_MeOH_ and its corresponding functions for tumour therapy.
