# Supplementary material for: Emerging integrated nanoclay-facilitated drug delivery system for papillary thyroid cancer therapy
Source: Sci Rep. 2016 Sep 12;6:33335. doi: 10.1038/srep33335 (PMC5018840; doi:10.1038/srep33335)
Supplement: Supplementary Information [file srep33335-s1.pdf]

Supplementary Information

Emerging integrated nanoclay-facilitated drug delivery system for papillary thyroid cancer therapy

Yi Zhang[1,†], Mei Long[1,†], Peng Huang[2,†], Huaming Yang[1,3,4,*], Shi Chang[2,*], Yuehua Hu[1,3], Aidong Tang[5], Linfeng Mao[2]

[1] Centre for Mineral Materials, School of Minerals Processing and Bioengineering, Central South University, Changsha 410083, China

[2] Xiangya Hospital, Central South University, Changsha 410078, China

[3] Hunan Key Lab of Mineral Materials and Application, Central South University, Changsha 410083, China

[4] State Key Lab of Powder Metallurgy, Central South University, Changsha 410083, China

[5] School of Chemistry and Chemical Engineering, Central South University, Changsha 410083, China

[*] Corresponding author, Email: H.Y.(email: hmyang@csu.edu.cn) or S.C.(email: changshi@csu.edu.cn), Fax: +86-731-88830549, Tel.: +86-731-88710804

[†] These authors contributed equally to this work.

Supplementary movie, tables and figures

Movie 1 The supplementary movie 1 clearly shows the passive targeting research *in vivo* (Pig)

Table S1 The loading amount, loading efficiency and cumulative release of DOX in DOX-Kaolin and DOX-modified-Kaolin

Table S2 Parameters of Korsmeyer−Peppas Model of cumulative release of DOX in DOX-Kaolin and DOX-modified-Kaolin

Figure S1 (a) XRD patterns, FTIR spectra, (c) zeta curves and (d) static water contact angle of the kaolin samples.

Figure S2 DAPI images of DOX, DOX-Kaolin$_{MeOH}$ and KI@DOX-Kaolin$_{MeOH}$ at different concentrations

Figure S3 CLSM images of thyroid cancer cells after the uptake of free DOX, Kaolin$_{MeOH}$, DOX-Kaolin$_{MeOH}$, KI@DOX-Kaolin$_{MeOH}$

Figure S4 (a) Inhibition of migratory potential of papillary thyroid cancer cells by wound healing assay before and after treatment with free DOX. (b) The percentages of migrated ($*P < 0.05$) and (c) invasive ($*P < 0.05$) cells determined by the migration and invasion assays

Figure S5 MTS assay and high-content screening of DOX-Kaolin$_{MeOH}$ with representative cancer cells

Table S1 The loading amount, loading efficiency and cumulative release of DOX in DOX-Kaolin and DOX-modified-Kaolin

Sample	Loading amount of DOX (%)	Loading efficiency (%)	Cumulative release(pH=4.5, 31 h)
Kaolin	54.41	90.69	43.84
$Kaolin_{MeOH}$	54.52	90.86	35.87

Table S2 Parameters of Korsmeyer–Peppas Model of cumulative release of DOX in DOX-Kaolin and DOX-modified-Kaolin

Sample	pH=4.5				pH=5.5				pH=7.4			
	n	k	b	R^2	n	k	b	R^2	n	k	b	R^2
Kaolin	0.46	9.67	-1.58	0.99	0.54	3.74	-1.19	0.96	0.39	1.15	-0.23	0.93
$Kaolin_{MeOH}$	0.42	9.05	-1.3	0.98	0.55	3.03	-0.86	0.91	0.58	0.59	0.262	0.87

$M_t/M_\infty = kt^n + b$

k is a constant that incorporates the structure and geometric characteristics of the drug dosage form, n is the release exponent characteristic of the release mechanism, and b represents the burst effect in the release.

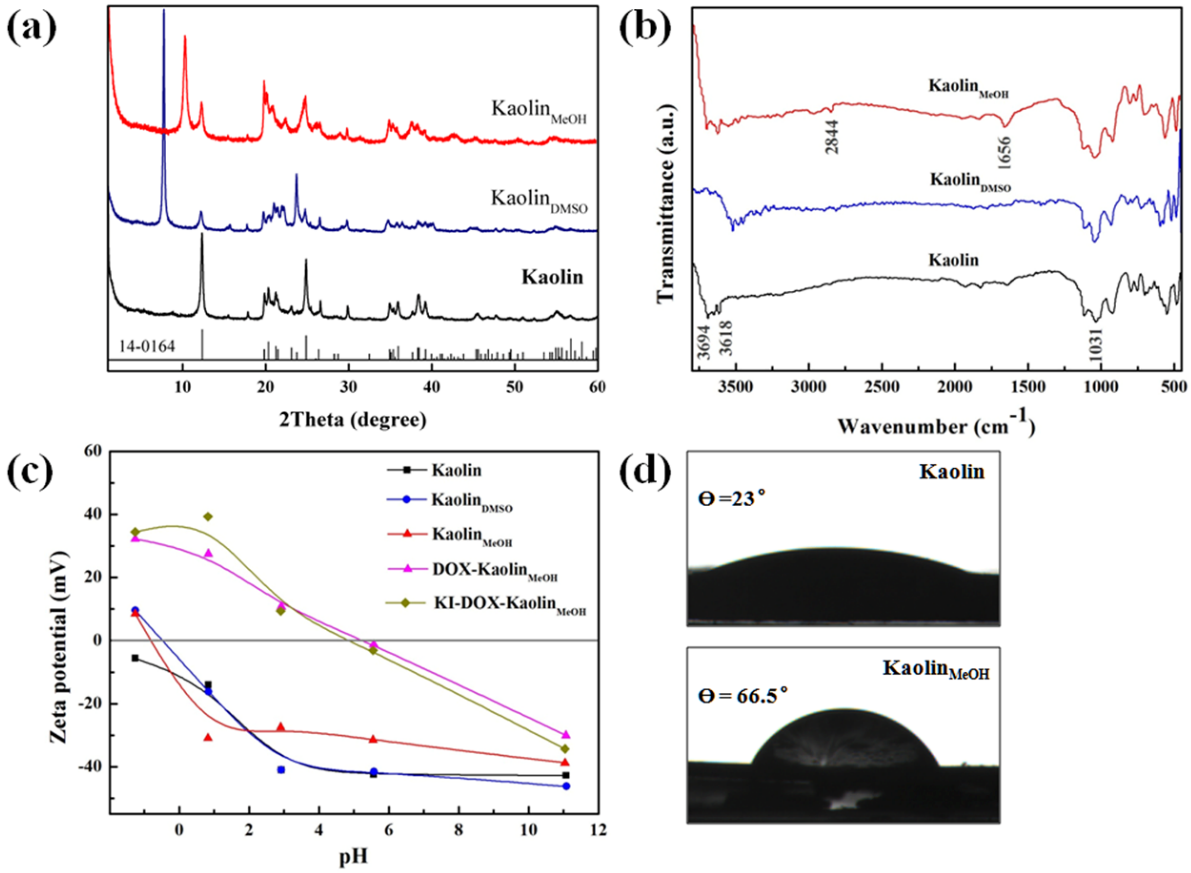

Figure S1 (a) XRD patterns, (b) FTIR spectra, (c) zeta curves and (d) static water contact angle of the kaolin samples.

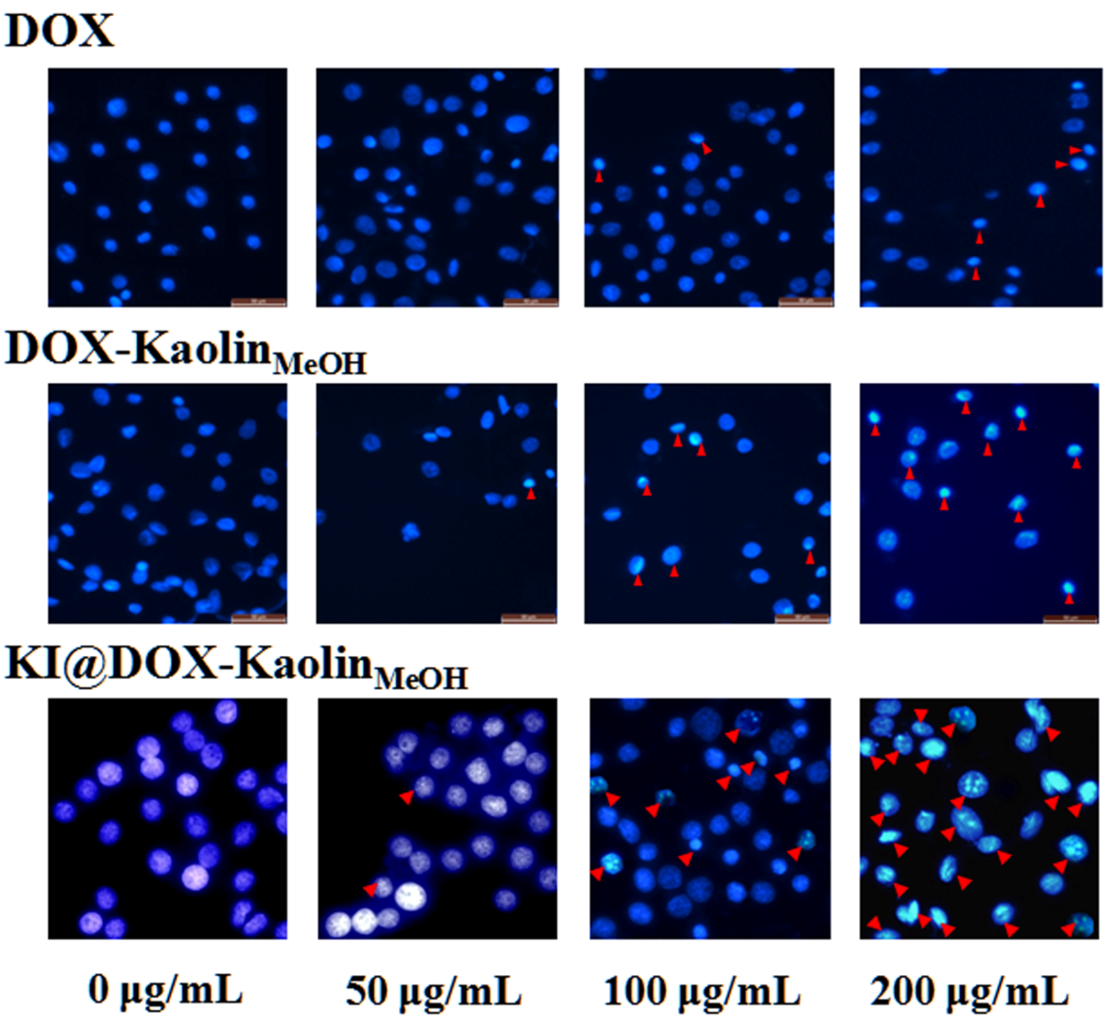

Figure S2 DAPI images of DOX, DOX-Kaolin$_{MeOH}$ and KI@DOX-Kaolin$_{MeOH}$ at different concentrations

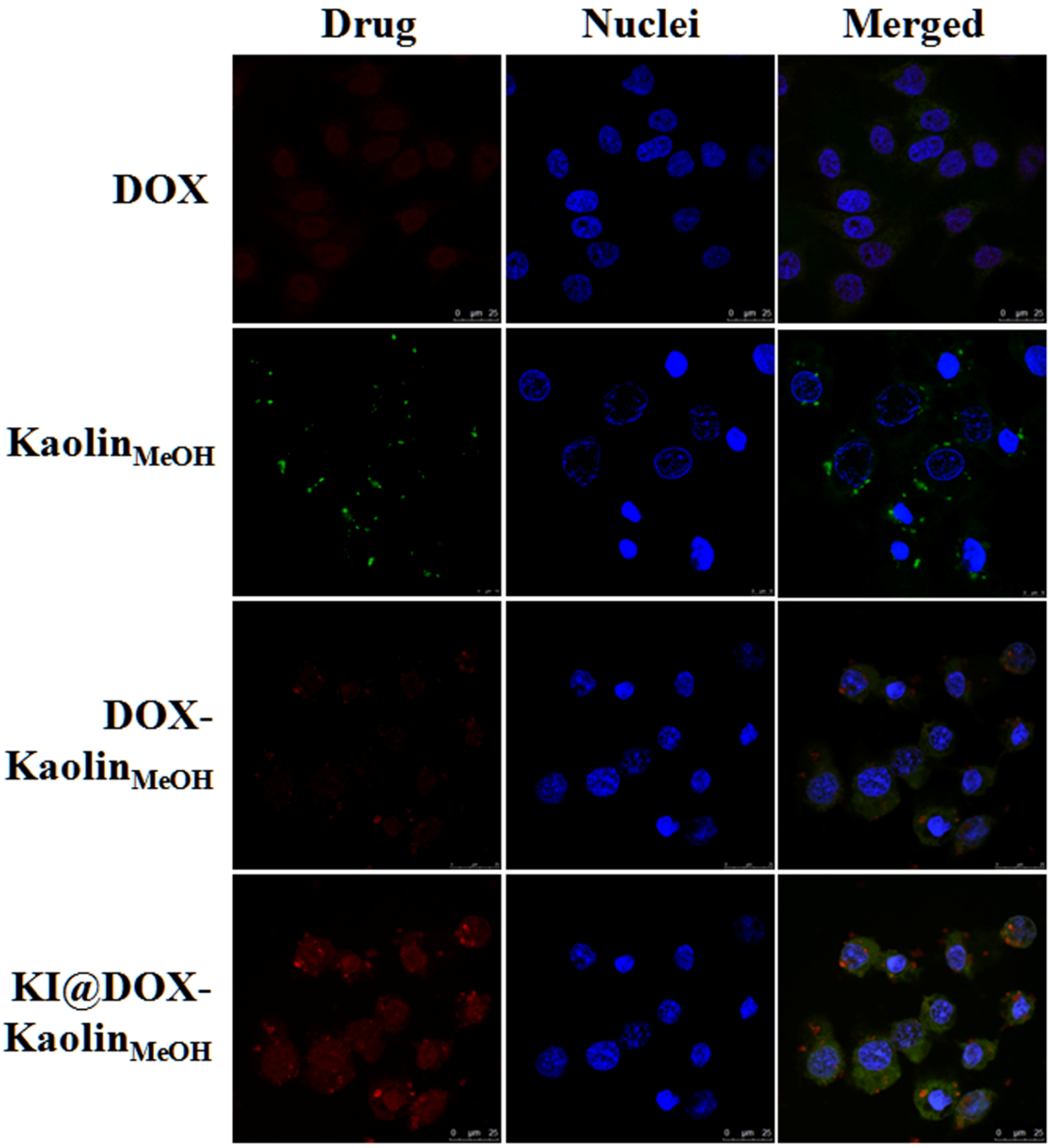

Figure S3 CLSM images of thyroid cancer cells after the uptake of free DOX, Kaolin$_{MeOH}$, DOX-Kaolin$_{MeOH}$, KI@DOX-Kaolin$_{MeOH}$

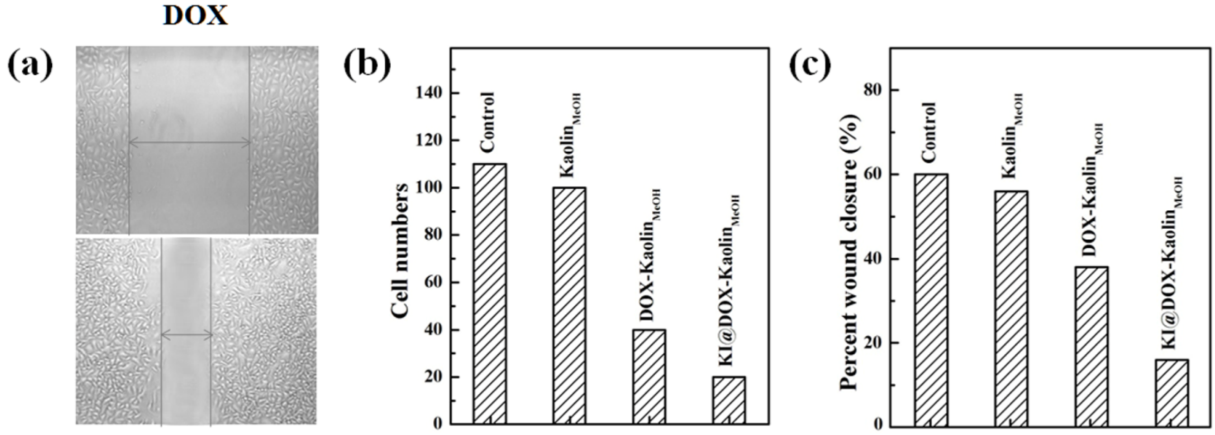

Figure S4 (a) Inhibition of migratory potential of papillary thyroid cancer cells by wound healing assay before and after treatment with free DOX. (b) The percentages of migrated ($*P < 0.05$) and (c) invasive ($*P < 0.05$) cells determined by the migration and invasion assays

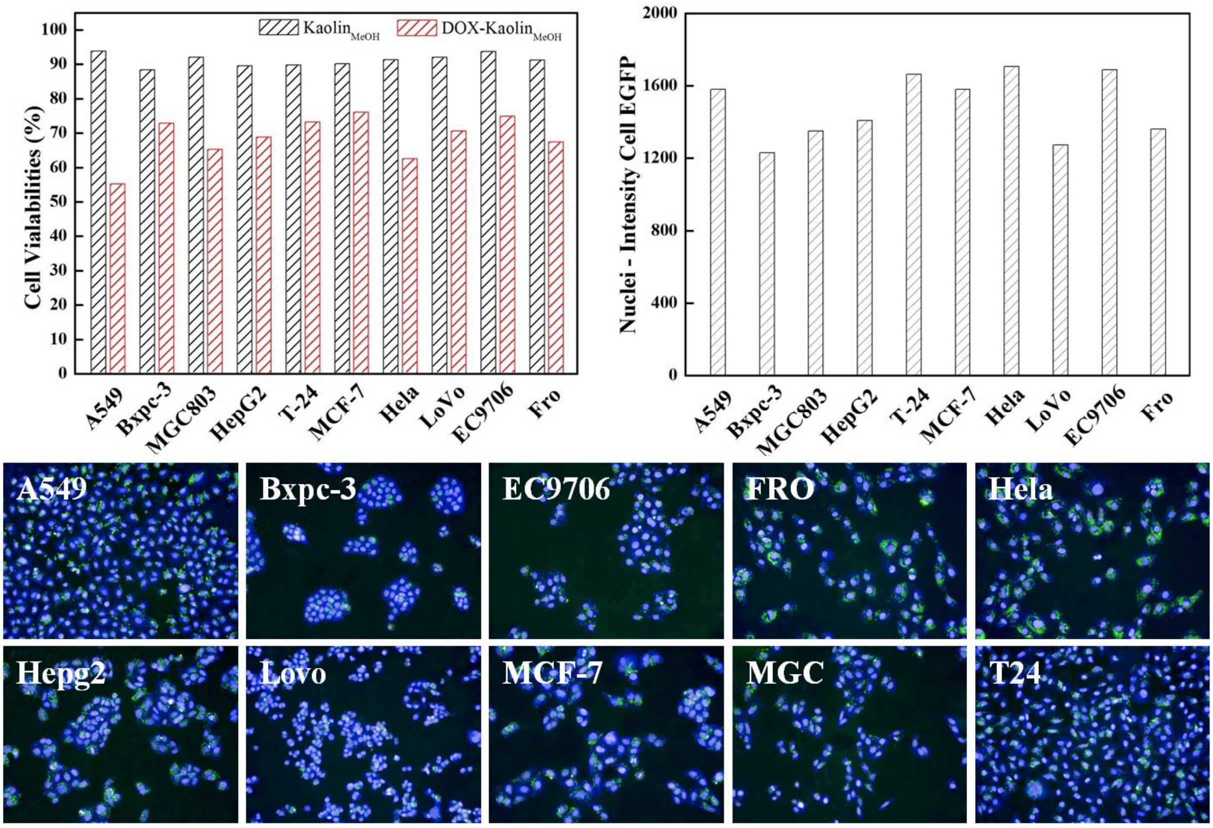

Figure S5 MTS Assay and High-Content Screening of DOX-Kaolin$_{MeOH}$ with representative cancer cells
